# Efficient RNA-guided base editing for disease modeling in pigs

**DOI:** 10.1038/s41421-018-0065-7

**Published:** 2018-12-18

**Authors:** Zhifang Li, Xiaoyue Duan, Xiaomeng An, Tao Feng, Pan Li, Linlin Li, Jun Liu, Panxue Wu, Dengke Pan, Xuguang Du, Sen Wu

**Affiliations:** 1 State Key Laboratory of Agrobiotechnology, College of Biological Sciences, China Agricultural University, Beijing, 100193 China; 2 College of Veterinary Medicine, China Agricultural University, Beijing, 100193 China; 3 Beijing Advanced Innovation Center for Food Nutrition and Human Health, China Agricultural University, Beijing, 100193 China; 4 State Key Laboratory of Veterinary Etiological Biology, National Foot and Mouth Diseases Reference Laboratory, Key Laboratory of Animal Virology of Ministry of Agriculture, Lanzhou Veterinary Research Institute, Chinese Academy of Agricultural Sciences, Lanzhou, 730000 China; 5 Institute of Organ Transplantation, Sichuan Academy of Medical Science & Sichuan Provincial People’s Hospital, Chengdu, 610072 China

Dear Editor,

Point mutations are the cause of many human genetic diseases^1^. However, the typical indel mutations generated by CRISPR/Cas9 make this system not ideal for modeling point mutations. The recently developed base editors such as the third generation base editor (BE3) have proved effective for creating missense mutations and early stop codons^2,3^, and have been used in zebrafish, mice, and rabbits through zygote microinjection^4–7^. In this study, we demonstrated the feasibility of BE3 and somatic cell nuclear transfer (SCNT) in generating pig models.

To test BE3 in pig fibroblasts, we chose *TWIST2* and *TYR* genes to examine the efficiency of base conversion (Fig. 1a–c). The one base change (amino-acid change) in the *TWIST2* gene is responsible for the ablepharon macrostomia syndrome (AMS) in human, resulting in severe deformities such as macrostomia, microtia, and absent eyelids^8^. The *TYR* gene is the causal gene for oculocutaneous albinism type 1 (OCA1)^9^.

**Fig. 1:**
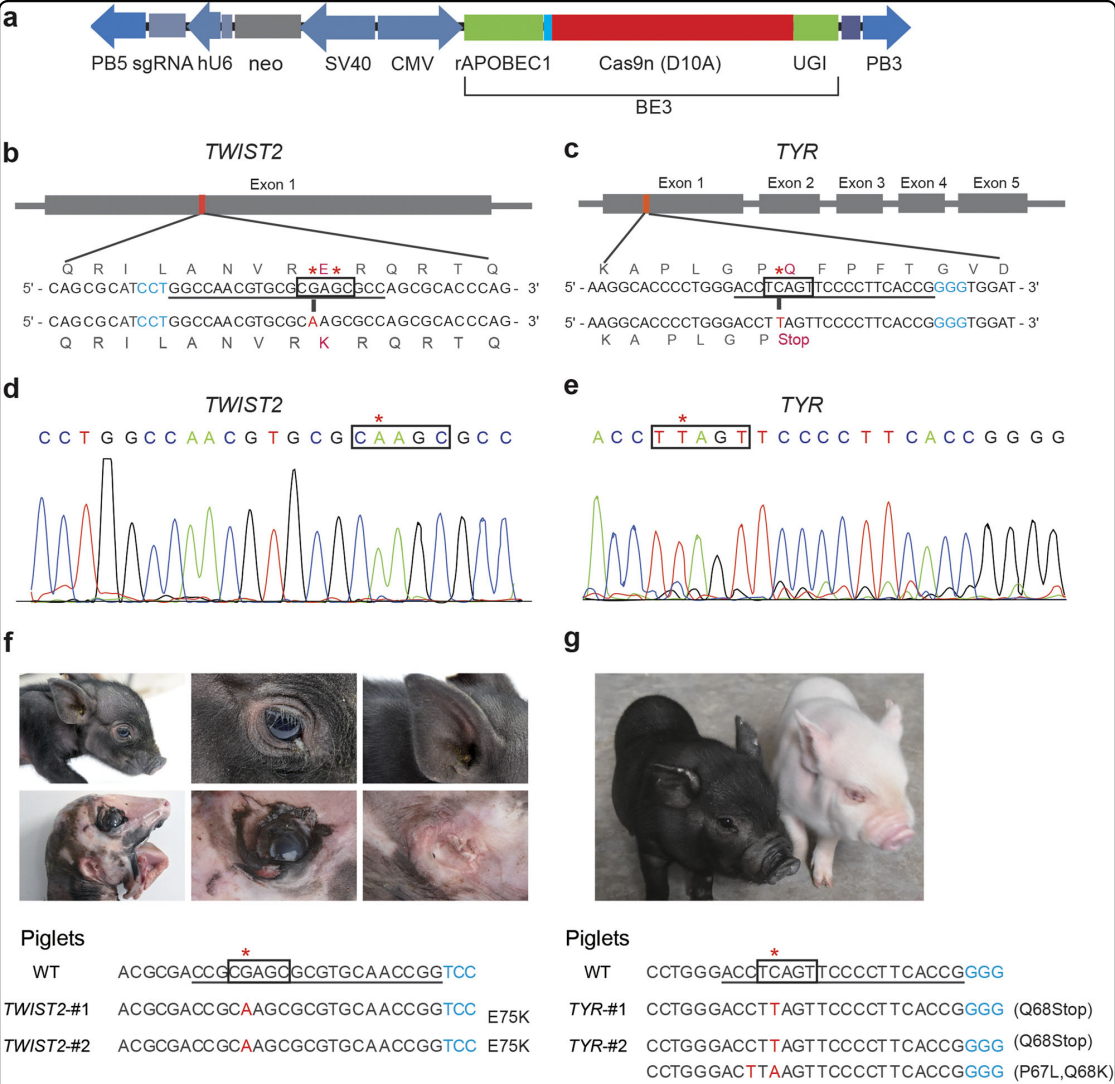
Efficient creation of pig models by BE3. **a** Schematic of the PB-BE3 vector. CMV cytomegalovirus promoter, rAPOBEC1 rat cytidine deaminase apolipoprotein B editing complex 1, Cas9n (D10A) Cas9 nickase with D10A mutation, UGI Uracil DNA glycosylase inhibitor, SV40 SV40 promoter, *neo* *neomycin* resistant gene, hU6 human U6 promoter, sgRNA single guide RNA, PB3 and PB5 terminals of the *piggyBac* transposon. **b**, **c** The target sequence at the *TWIST2* and *TYR* loci. Missense (E75K) and nonsense (Q68Stop) mutations were introduced by BE3 in *TWIST2* and *TYR* gene, respectively. The base editing window of BE3 is approximately 5 nt, typically -17 to -13 upstream of the PAM sequence. The asterisk represents the target site. The PAM sequence is shown in cyan. The sgRNA target sequence is underlined. The nucleotides and amino acids substituted by BE3-mediated base editing are shown in red and magenta. **d**, **e** Representative sequencing chromatograms of porcine fetal fibroblast clones at the *TWIST2* and *TYR* loci. The relevant codons are underlined in red. **f** Compared with wild-type newborn piglet (top panel), *TWIST2* mutant piglets (middle panel) exhibit severe deformities such as macrostomia, microtia, and absent eyelids. Representative genotypes of *TWIST2* mutant piglets are illustrated at the bottom panel. **g** The white piglet is *TYR* mutant exhibiting a typical albinism phenotype, compared with the black one on the left with a heterozygous mutation. Representative genotypes of *TYR* mutant piglets are illustrated at the bottom

After transfecting porcine fetal fibroblasts (PFFs) with plasmids expressing BE3 and sgRNA components (Supplementary Table S1), 43 clones for the *TWIST2* gene were picked and expanded. Among these, 36 clones were confirmed to be edited by BE3 with a high efficiency (84%). There are two C’s in the editing window of the sgRNA target site. Among the 36 clones, 15 carried two C to T substitutions, 21 carried one C to T substitution, and no clone was found to have C to T substitution outside the base editing window. In total, 11 clones were homozygous mutations carrying a glutamate to lysine amino-acid change (Fig. 1d, e; Supplementary Fig. S1a and Table S2), precisely mimicking the p.E75K mutation found in human. For the *TYR* gene, 66 clones were picked and expanded. There is only one C in the editing window of the sgRNA target site. Among the 66 clones, eight clones were introduced with an early stop codon, and one clone carried the homozygous mutations. Also, one clone was found to have C to T substitution outside the base editing window (Supplementary Fig. S1b).

Next, we performed SCNT to investigate the developmental capacity of these BE3-edited cells in vivo. In total, we obtained 21 piglets derived from the *TWIST2* clones (*TWIST2*-#22 and *TWIST2*-#54) containing the E75K mutations, and 4 piglets from the *TYR* clones (*TYR*-#1 and *TYR*-#8) containing the Q68Stop mutations (Supplementary Table S3). All of the *TWIST2* piglets were confirmed by sequencing to contain the same base conversion (E75K), and they showed expected phenotypes similar to human patients with absent eyelids, microtia, macrostomia, hypotrichosis, and abnormal trotters (Fig. 1f). In addition, *IL-1β*, which is normally inhibited by *TWIST2*, was found highly expressed in the skin by qRT-PCR (Supplementary Fig. S2a), consistent with previous study^10^. Among the four *TYR* piglets, three were carrying homozygous mutations (Q68Stop), and one had an allele of Q68Stop and an allele of 15 bp deletion. These four piglets showed typical albinism phenotypes and completely lost dark pigment in skin, hair, and eyes (Fig. 1g). Western blot showed that tyrosinase was not expressed in the heart, liver, lungs, or kidney (Supplementary Fig. S2b). In addition, we examined ten potential off-target sites for *TYR* and *TWIST2* sgRNAs, and no off-target mutations were detected in the mutant piglets (Supplementary Fig. S3, Table S4 and S5). The above results indicate that the base editing system is successful in generating pig models (Table 1).

**Table 1:** Summary of base editing rate in porcine fetal fibroblasts and offspring using BE3

Target gene	No. of clones	No. of mutants (%)	No. of heterozygote (%)	No. of homozygote (%)	No. of indel (%)	No. of offspring
*TWIST2 TYR*	4366	36 (84)8 (12)	25 (58)7 (11)	11 (26)1 (2)	1 (2)2 (3)	218

To examine the efficiency of BE3 in editing multiple copies of genes, we selected the porcine endogenous retroviruses (PERVs), which have various copy numbers among different pig breeds. According to droplet digital PCR, the PERVs copy number of the pig fibroblast lines we used in this study was 34, 33, and 30, respectively (Supplementary Fig. S4a). Two sgRNAs were used to target the highly conserved enzyme activity center of the *pol* gene of PERVs to create premature termination codons. We picked and analyzed 326 pig fibroblast clones modified by BE3. By deep sequencing, we found that ~13.3% of clones were edited for more than six copies simultaneously, and up to 20 copies of PERVs could be edited (Supplementary Fig. S4b, c and Table S6). In addition, we compared the toxicity of BE3 with Cas9 for editing PERVs in cell culture. Using the immunofluorescent staining of Phospho-Histone H2A.X and Annexin V, we demonstrated that fewer early apoptotic cells were produced by BE3 than Cas9, and more live cells were present in the BE3 group (Supplementary Fig. S5). The above results suggest that BE3 may be a safer tool for modifying multiple genes simultaneously.

In summary, we confirmed that the BE3 system could achieve C-to-T (G-to-A) conversions in cell culture both for individual genes and multiple copies of genes (up to 20). The pig models created via BE3 closely reproduced the phenotypes of human diseases. Besides, a genome-wide analysis showed that early stop codons could be introduced by BE3 in 16,677 pig genes (Supplementary Fig. S6 and Table S7), suggesting a broader range of potential applications of this technology. With the development of new technologies, which have wider PAM compatibility, even more genes could be base edited^11^.

Consistent with previous studies, our observations found that the double strand DNA break (DSB) resulted from Cas9 could cause DNA damage and cell death, especially in the case of editing multiple copies of genes^3^. In contrast, BE3 creates termination codons in the open reading frame of PERVs without DSB damage. Although there are some unwanted cytosine changes in or outside the editing window, different variants of cytidine deaminases can be selected to narrow the editing window to further improve the precision of base editing^12,13^. Finally, through combination of nuclear transfer and base editing, homozygous or heterozygous mutant cells can be conveniently selected in vitro, ensuring precise model creation. In conclusion, base editing systems provide a safer and more efficient approach to generate pig models that can precisely mimic mutations of human diseases.

## Electronic supplementary material

Supplementary Information

Supplementary Table S7

## Electronic supplementary material

**Supplementary Information** accompanies the paper at (10.1038/s41421-018-0065-7).
