# Supplementary material for: Efficient RNA-guided base editing for disease modeling in pigs
Source: Cell Discov. 2018 Dec 18;4:64. doi: 10.1038/s41421-018-0065-7 (PMC6297129; doi:10.1038/s41421-018-0065-7)
Supplement: Supplementary file 1 — Supplementary Information [file 41421_2018_65_MOESM1_ESM.pdf]

Supplemental Information for

Efficient RNA-guided base editing for disease modeling in pigs

This PDF file includes:

Supplementary M&M

Supplementary Figs. S1-S6

Supplementary Tables. S1-S6

Supplementary M&M

Ethics statements. Procedures with animals were approved by the Animal Welfare Committee of China Agricultural University SKLAB-2017-04-01.

sgRNA design and plasmids construction. sgRNAs were designed with the help of the website: http://www.rgenome.net/be-designer. The targeting sites of PERVs were in the highly conserved enzyme active region of the pol gene. The target sequences of PERVs were confirmed by deep sequencing of the WT cells using the degenerate primers. We designed two sgRNAs in a relatively conserved region that could generate an early stop codon after successful base conversion. The sgRNA sequences were shown in Supplementary Table S1. To build a plasmid that contains both BE3 and sgRNA, the original vector pCMV-BE3, purchased from Addgene (plasmid #73021), was digested with restriction enzyme PmeI/MluI as the backbone. Then U6-sgRNA expression cassette from pX330 (Addgene plasmid #42230), SV40-neo from pIRES2-EGFP (Clontech), and the PB 5′ and 3′ terminals from the pZGs vector were amplified and assembled into the backbone. The resulting plasmid is named PB-BE3 (Supplementary Table S1).

Quantitative RT-PCR. Total RNA was isolated using RaPure Total RNA Kit (Magen). Reverse transcription was performed using TransScript One-Step gDNA Removal and cDNA Synthesis SuperMix (TRANS). For quantitative RT-PCR, 2×RealStar Green Fast Mixture (GenStar) was used, and the ABI 7300 system was used to collect signal (Primer sequences see Supplementary Table S1).

Western blot. The tissues (heart, liver, lungs, and kidney) from the wild-type and *TYR* Q68stop mutant piglets were lysed in RIPA lysis buffer (Beyotime) and homogenized by

TissueLyser LT (Qiagen) for 5 minutes. The tissue lysates were kept on the ice for 30 minutes and then centrifuged at 14,000 g for 10 minutes at 4°C. The total protein was measured with BCA Protein Assay Kit (Beyotime) and the protein samples were denatured at 99°C for 10 minutes. Twenty μg total protein were separated by 10% SDS-PAGE and transferred onto Immobilon-P transfer membrane (Millipore). After blocking with 5% nonfat dry milk, membranes were incubated with primary antibodies against tyrosinase (1:800, rabbit polyclonal, Abcam) and GAPDH (1:10,000, rabbit monoclonal, Thermo) at 4°C overnight. After washing with PBST and incubated with HRP labeled goat-anti-rabbit IgG secondary antibody (1:1,000, Beyotime) at room temperature for 1 hour, the membranes were washed again and incubated with SuperSignal West Dura Extended Duration Substrate (Thermo) according to the manufacturer's instructions. Finally, the membranes were exposed to X-ray film (Fuji film).

Droplet Digital PCR (ddPCR). We used ddPCR to detect the copy number of PERVs. Genomic DNA isolated from cell lines was digested with MseI and used as the ddPCR template. The PCR procedure was performed according to the manufacturers' instructions of QX200™ Droplet Digital™ PCR system (Bio-Rad). The primers and the probes used in the ddPCR were listed in Supplementary Table S6.

Off-target assay. Ten potential off-target sites for each sgRNA were predicted using Cas-OFFinder.[1] The corresponding PCR products were sequenced. All primers for off-target assay are listed in Supplementary Table S5.

Targeting efficiency calculation. We used the targeted deep sequencing to estimate the targeting efficiency of each clone. Each primer was added a different barcode sequence. We

amplified the target region with different barcode primer pairs. Amplicons of 15 samples were mixed and sequenced as one library using Illumina PE150. The sequence data of each sample were separated by the barcodes. Since there are three potential sites where an early stop codon could be introduced with the two sgRNAs (PERV sgRNA5 and PERV sgRNA6), if >=1 site was targeted as C-T conversion, this copy of PERV was defined as targeted. Next, we calculated the targeting efficiency as the percentage of targeted reads in total alignment reads and calibrated this efficiency with the WT data. Finally, we verified the targeted sequences using TA cloning and Sanger sequencing, which was consistent with the deep sequencing results.

Immunofluorescent staining. Twenty-four hours after transfection, the cells were digested with 0.1% trypsin (Invitrogen), and centrifugated for 5 minutes at 180 g. The pelleted cells were used for Immunofluorescent staining of Phospho-Histone H2A.X and Annexin V (cat. nos. 2577 and 6592, Cell signaling) according to the manufacturer's instructions.

Cell culture, transfection, and selection. Primary porcine fetal fibroblasts (PFFs) were isolated from 30 days-old fetuses from strains called Chinese experimental miniature swine (CEMS). The PFFs were cultured in Dulbecco's modified Eagle's medium (DMEM, Invitrogen) containing 10% fetal bovine serum (FBS, Gibco), 1% nonessential amino acids (Invitrogen), 1% penicillin-streptomycin (Gibco). Corresponding 4.5 μg PB-BE3-sgRNA plasmid and 1.5 μg CAG-PBase were transfected into 10^6 PFFs using a Nucleofector™ 2b Device (Lonza) with program A-024. Then, cells were plated on 6-well dish. After 24 hours of culture, cells were transferred to twenty 10-cm dishes for drug selection with 800 μg/ml G418 (Gibco) for two weeks. The picked clones were sequenced and analyzed.

In vitro maturation of oocytes. Methods used for porcine oocytes collection, in vitro maturation were similar to our previous study.[2] Briefly, pig ovaries were collected from a local abattoir and transported to the laboratory within 3 hours in 0.9% NaCl at 35°C~38°C. Cumulus-oocyte complexes (COCs) were aspirated from medium-sized (3~6 mm) follicles with a gauge 12 needle fixed to a vacuum pumping system. The COCs were washed three times in maturation medium, and then 80 COCs per 500 μl were cultured in the maturation medium for 42~44 hours at 38.5°C. Matured COCs were digested with 0.1% hyaluronidase (Sigma H4272) to remove cumulus cells.

Nuclear transfer and embryo transfer. Nuclear transfer was performed as previously described.[2] In brief, cultured positive cells were used as donor cells. A single donor cell was injected into the perivitelline space of an enucleated oocyte. Oocyte cytoplasm-cell complexes were fused and activated using an electrofusion instrument CF-150Bsp (BLS). The reconstructed embryos were cultured in PZM-3 at 38.5°C, 5% CO_2 overnight, and were transferred into surrogate mothers. A month later, the pregnancy status of the surrogates was detected by ultrasonography. The piglets were born through natural birth.

Bioinformatics for genome-wide i-stop analysis. The i-stop (induction of STOP codons) targetable sites were identified from all candidate codons (CAA, CAG, CGA and TGG) that can potentially be converted to stop codons (TAA, TAG, and TGA) by BE3. Briefly, we search the locations of three amino acids (arginine, glutamine, tryptophan) in the pig genome, and verify the corresponding nucleotide sequences. We then select the sgRNAs that meet the standard of 5′-N(3 to 7) -(CGA/CAG/CAA)-N(14 to 10)- NGG(PAM) on the coding strand or 5′-N(2 to 7) -(CCA)-N(15 to 10)-NGG(PAM) on the noncoding strand.[3] The final i-stop

library contained 202,387 sgRNAs for 16,677 genes.

Reference

1. Bae, S., Park, J. & Kim, J. S. Cas-OFFinder: a fast and versatile algorithm that searches for potential off-target sites of Cas9 RNA-guided endonucleases. *Bioinformatics* **30**, 1473–1475 (2014).
2. Du, X. G. *et al*. Barriers for deriving transgene-free pig iPS cells with episomal vectors. *Stem Cells* **33**, 3228-3238 (2015).
3. Kuscu, C. *et al.* CRISPR-STOP: gene silencing through base-editing-induced nonsense mutations. *Nat Methods* **14**, 710-712 (2017).

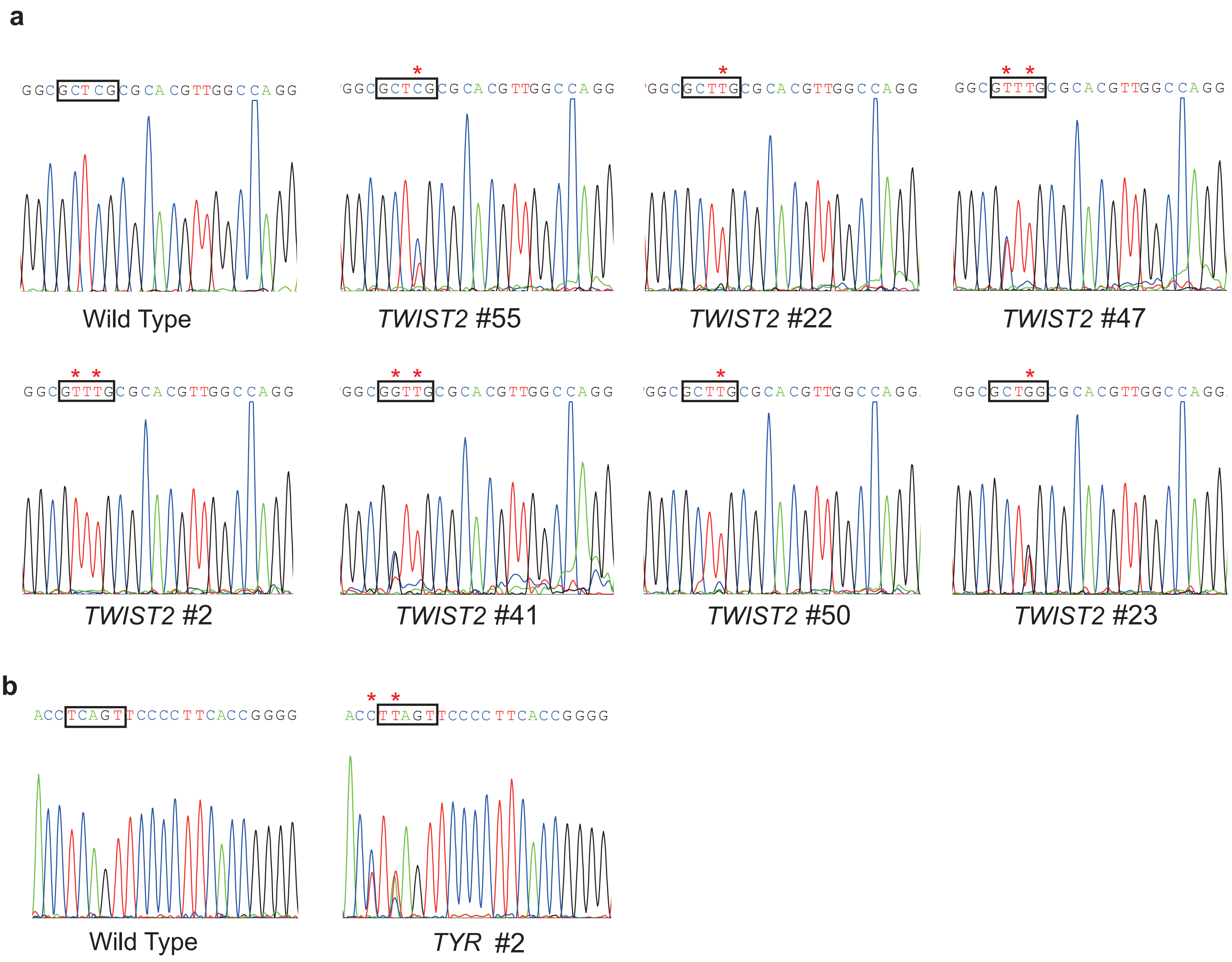
a
Wild Type
TWIST2 #55
TWIST2 #22
TWIST2 #47
TWIST2 #2
TWIST2 #41
TWIST2 #50
TWIST2 #23
b
Wild Type
TYR #2

Supplementary Figure S1. Summary of mutant cell clones

a Generation of *TWIST2* mutant clones using BE3.

b Generation of *TYR* mutant clones using BE3.

The base-editing window of BE3 is shown in black box. The asterisk is shown the target site.

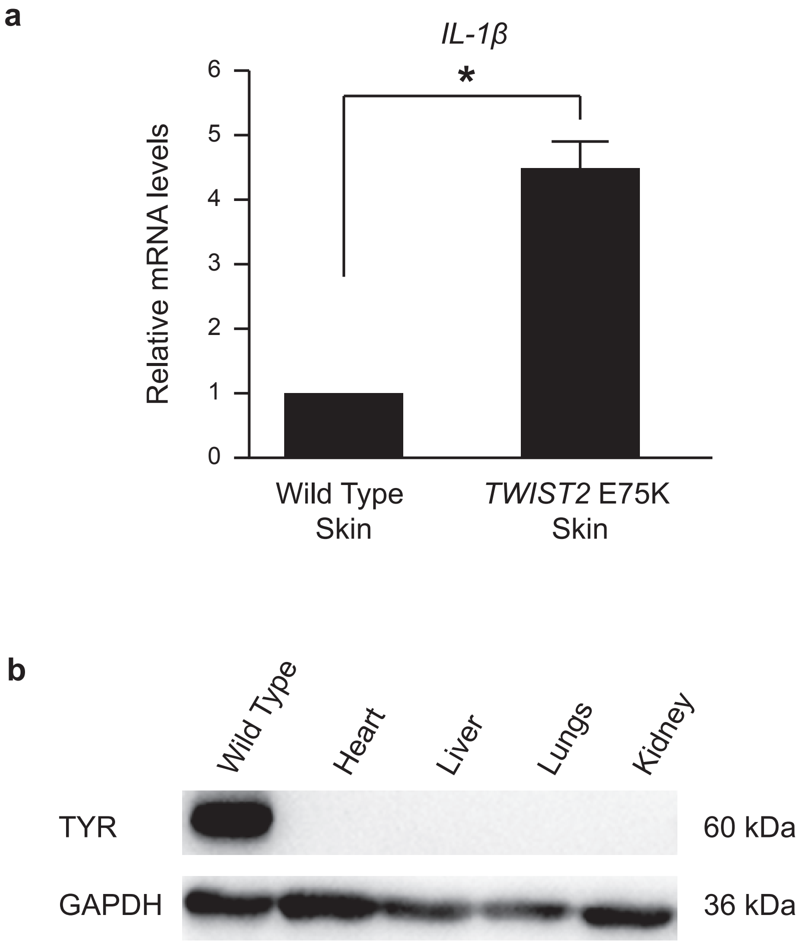
a
IL-1β
*
Relative mRNA levels
0
1
2
3
4
5
6
Wild Type Skin
TWIST2 E75K Skin
b
Wild Type
Heart
Liver
Lungs
Kidney
TYR
GAPDH
60 kDa
36 kDa

Supplementary Figure S2. Characterization of mutant piglets

a qRT-PCR for *IL-1β* in *TWIST2* E75K mutant piglets. Data are analyzed by Student's t test and are presented as mean ± SEM, $^{*}p < 0.05$.

b Western blot of TYR protein in *TYR* Q68stop mutant piglets.

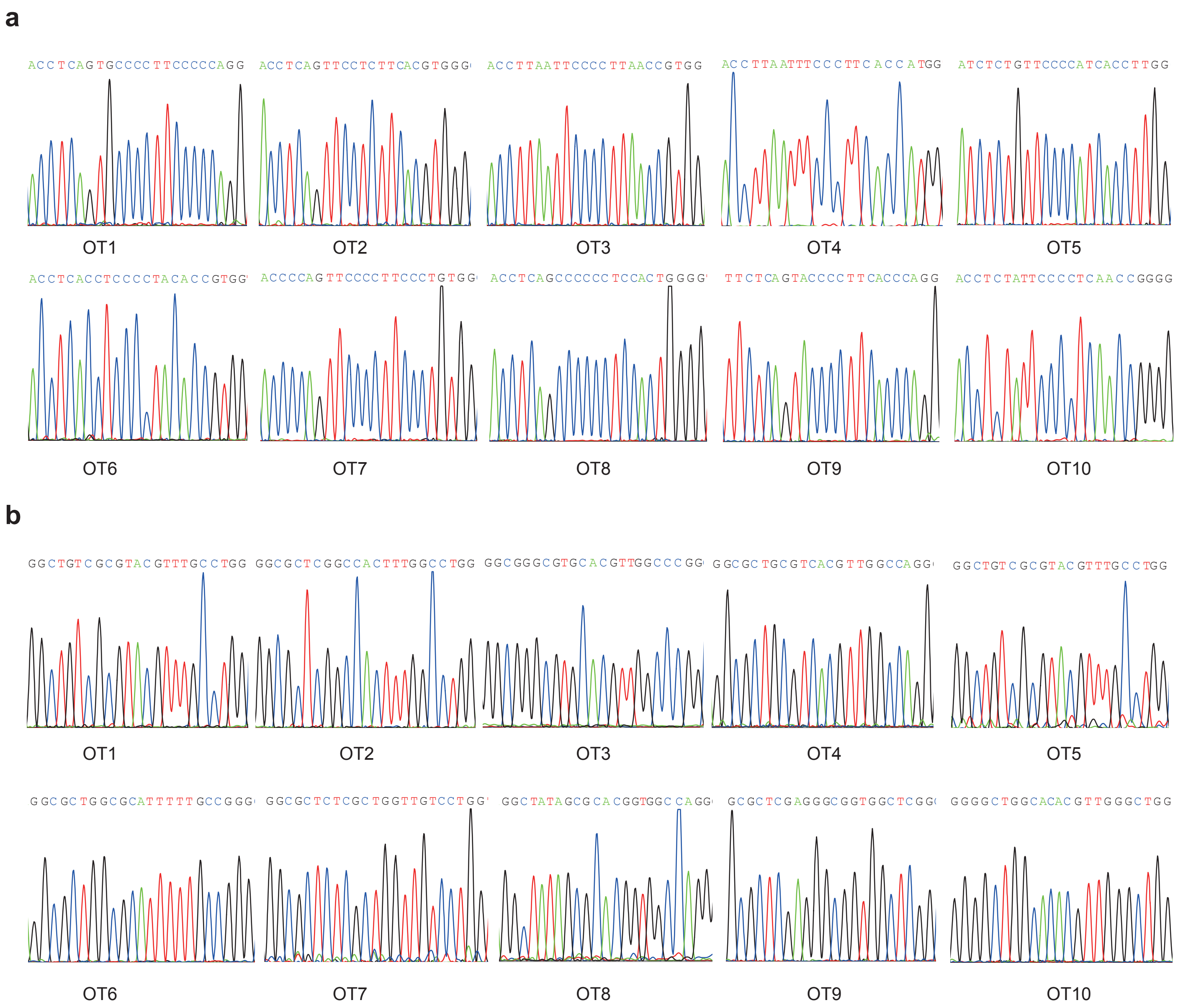
a
OT1
OT2
OT3
OT4
OT5
OT6
OT7
OT8
OT9
OT10
b
OT1
OT2
OT3
OT4
OT5
OT6
OT7
OT8
OT9
OT10

Supplementary Figure S3. Off-Target analysis in mutant piglets

Sequencing of 10 potential off-target sites for *TYR* and *TWIST2* sgRNAs using PCR products for *TYR* Q68Stop (a) and *TWIST2* E75K (b).

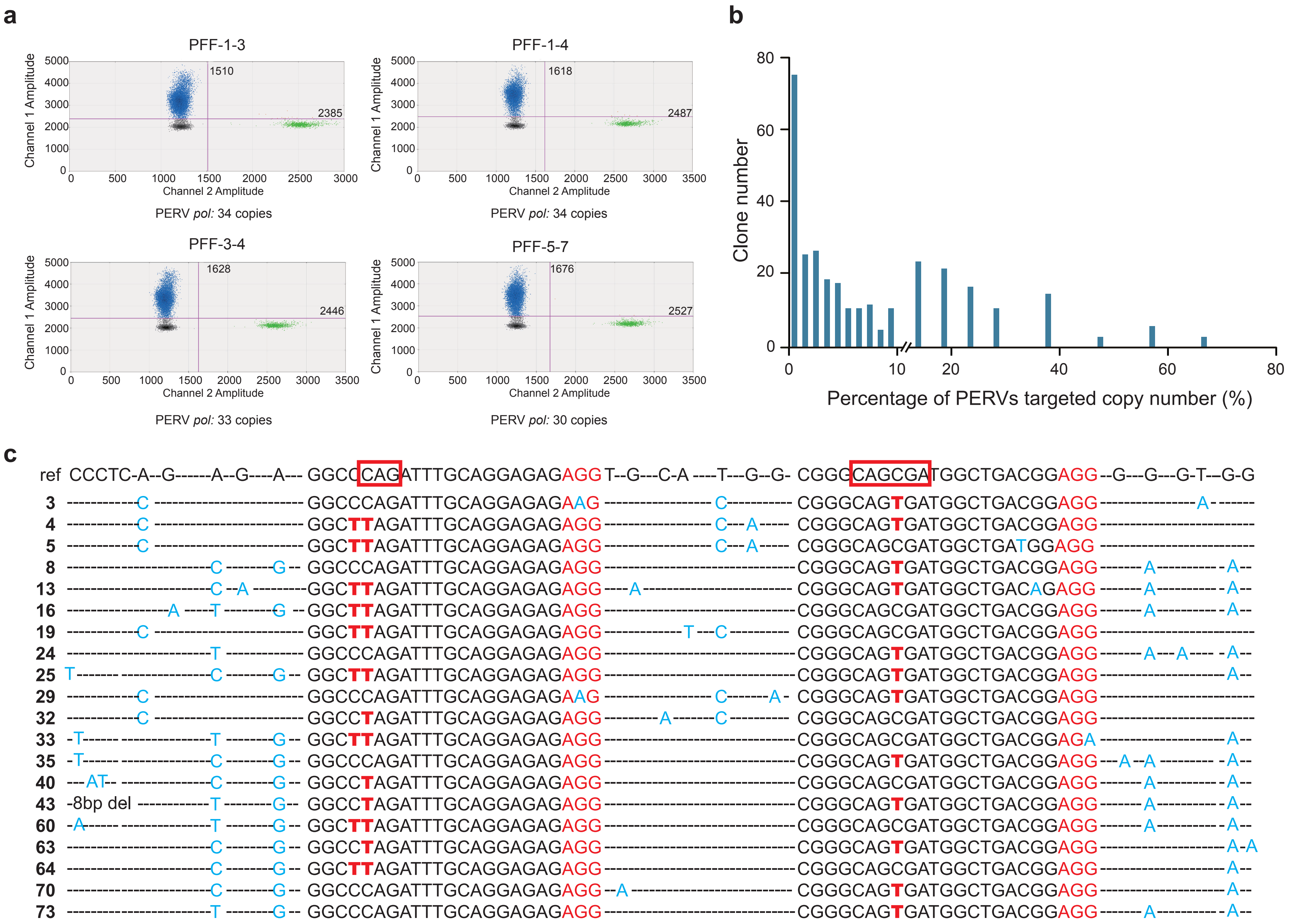
a
PFF-1-3
1510
2385
Channel 1 Amplitude
Channel 2 Amplitude
PERV pol: 34 copies
PFF-1-4
1618
2487
PERV pol: 34 copies
PFF-3-4
1628
2446
PERV pol: 33 copies
PFF-5-7
1676
2527
PERV pol: 30 copies
b
Clone number
Percentage of PERVs targeted copy number (%)
c
ref
-8bp del

Supplementary Figure S4. Multiple copies of PERVs in PFFs can be edited by BE3.

a Detection of copy number of PERVs in PFF cell lines using digital droplet PCR. In each 2D amplification graph, the gray droplets mean no amplification in the absence of templates, the blue droplets contain PERVs, the green droplets contain *GAPDH* gene (reference gene), and the orange droplets contain PERVs and *GAPDH* gene. The copy number of PERVs is calibrated by normalizing to the *GAPDH* gene.

b Calculation of targeting efficiency (%) of PERVs in 326 clones by high-throughput sequencing.

c Sequences of the 20 targeted copies of PERVs in one clone. The two target sites are shown in the reference sequence and in targeted sequences. The red letters are the protospacer adjacent motif (PAM). The bold red "T" bases are mutations caused by BE3. The red frames show the potential stop codon if base edited. The blue letters are SNPs that can distinguish among different copies of PERVs. The numbers in the left column represent different reads via high-throughput sequencing.

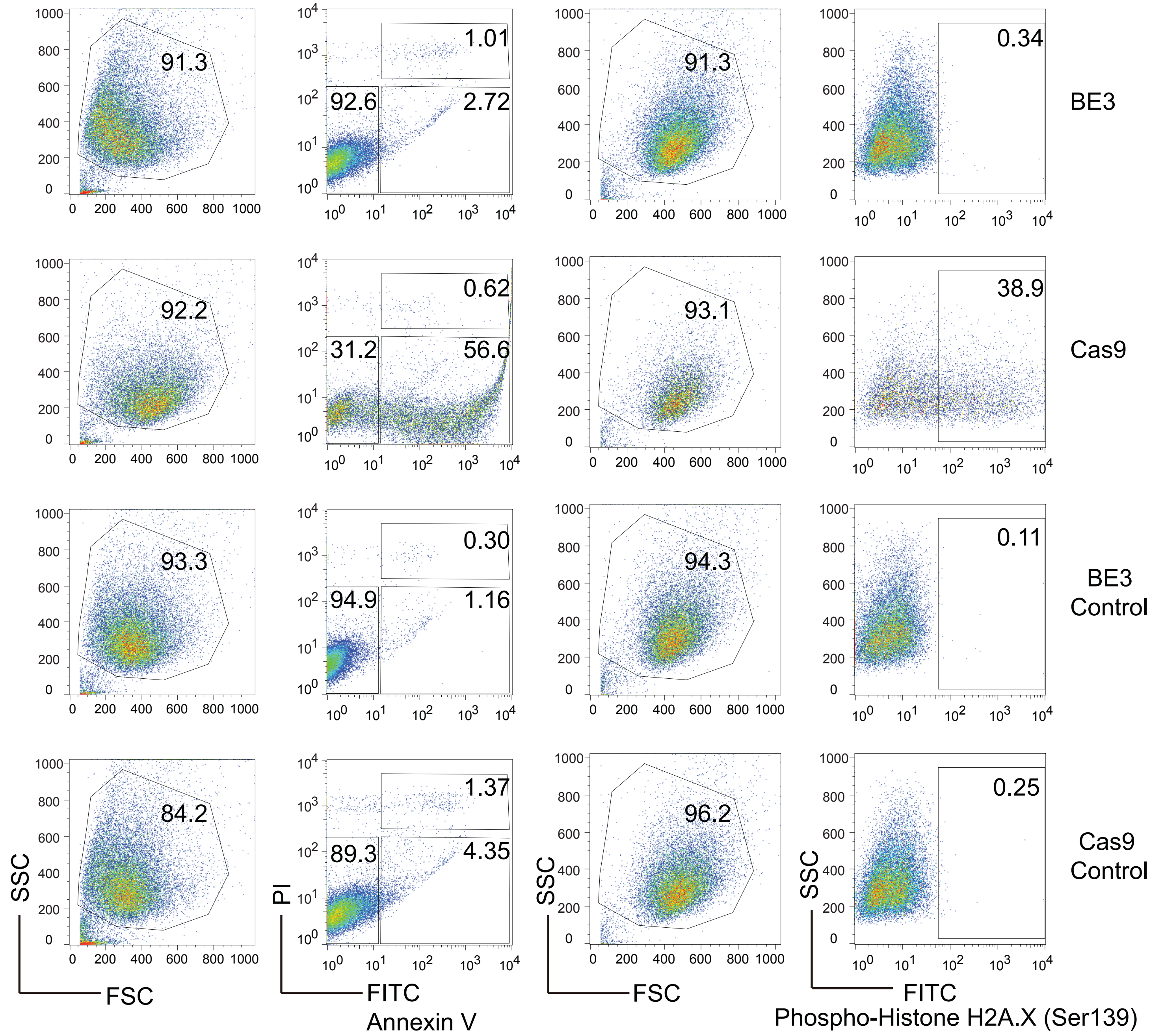

BE3
Cas9
BE3 Control
Cas9 Control
91.3
1.01
92.6
2.72
91.3
0.34
92.2
0.62
31.2
56.6
93.1
38.9
93.3
0.30
94.9
1.16
94.3
0.11
84.2
1.37
89.3
4.35
96.2
0.25
SSC
FSC
PI
FITC
Annexin V
SSC
FSC
SSC
FITC
Phospho-Histone H2A.X (Ser139)

Supplementary Figure S5. BE3 is less toxic than Cas9 in editing PERVs.

The flow cytometric plots show immunofluorescent staining of Annexin V and Phospho-Histone H2A.X respectively after transfection. The BE3 control and Cas9 control groups were transfected with the same plasmids as the experimental groups but without the sgRNAs.

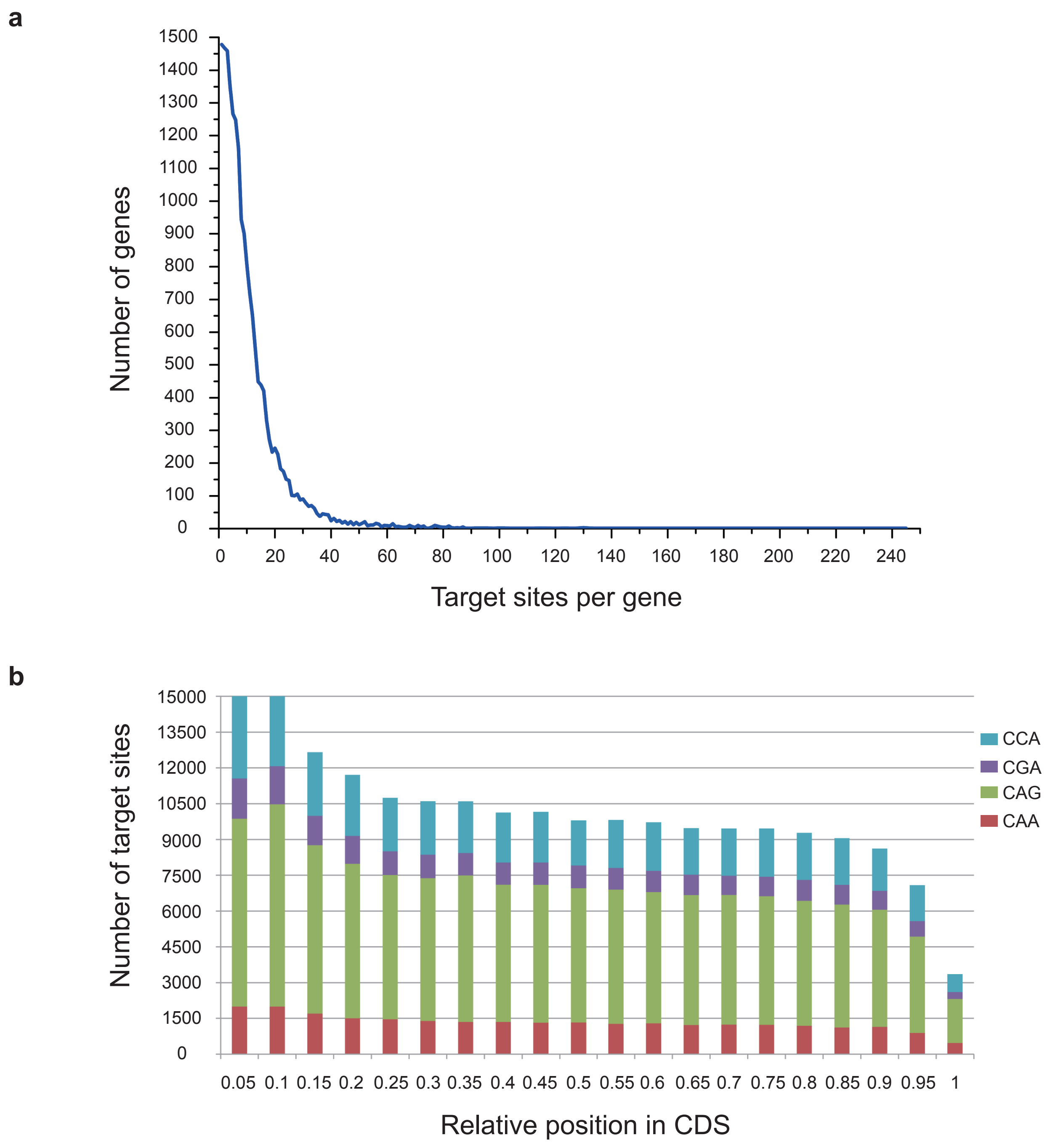
a
Number of genes
1500
1400
1300
1200
1100
1000
900
800
700
600
500
400
300
200
100
0
0 20 40 60 80 100 120 140 160 180 200 220 240
Target sites per gene
b
Number of target sites
15000
13500
12000
10500
9000
7500
6000
4500
3000
1500
0
0.05 0.1 0.15 0.2 0.25 0.3 0.35 0.4 0.45 0.5 0.55 0.6 0.65 0.7 0.75 0.8 0.85 0.9 0.95 1
Relative position in CDS
CCA
CGA
CAG
CAA

Supplementary Figure S6. i-stop sgRNAs are widely distributed in the pig genome.

a The number of targetable genes with different sgRNA(s) is shown.

b Relative positions of i-stop sites in the coding sequences (CDSs). Four targetable codons are shown in different colors.

Supplementary Table S1. List of primers

Primer Name	Primer sequence
**sgRNA sequence**	
AXM-TYR-sgRNA-S	CACCGACCTCAGTTCCCCTTCACCG
AXM-TYR-sgRNA-A	AAACCGGTGAAGGGGAACTGAGGTC
AXM-TWIST2-sgRNA-S	CACCGGCGCTCGCGCACGTTGGCC
AXM-TWIST2-sgRNA-A	AAACGGCCAACGTGCGCGAGCGCC
PERV-sgRNA-BE3-5S	CACCGGGCCCAGATTTGCAGGAGAG
PERV-sgRNA-BE3-5A	AAACCTCTCCTGCAAATCTGGGCCC
PERV-sgRNA-BE3-6S	CACCGCGGGCAGCGATGGCTGACGG
PERV-sgRNA-BE3-6A	AAACCCGTCAGCCATCGCTGCCCGC
**PCR primers used for amplifying fragments around target sites**	
AXM-TYR-421-F	GCTCAATTAACAAGCTCAAACAGA
AXM-TYR-421-R	ATCAAAGATGTTTCTTCTGACCAA
AXM-TYR-329-F	GCTTTGTACTGCCTGCTCTGGACT
AXM-TYR-329-R	TCTGTGCAGTTGGGTCCCTGAAAG
AXM-TWIST2-250-F	CTACAGCAAGAAGTCGAGCGAAGA
AXM-TWIST2-250-R	GAGGAAGTCTATGTACCTGGCGGC
**Primers of quantitative RT-PCR**	
AXM-qPCR-pig-GAPDH-F	ATCACCATCTTCCAGGAGCGA
AXM-qPCR-pig-GAPDH-R	AGCCTTCTCCATGGTCGTGAA
AXM-qPCR-IL1-126-F1	TAGTACCTGAACCCGCCAAG
AXM-qPCR-IL1-126-R1	TCAGAGAACCAAGGTCCAGG
**Primers and probes of ddPCR**	
Pol-F1	CGACTGCCCCAAGGGTTCAA
Pol-F2	CCGACTGCCCCAAGAGTTCAA
Pol-R	TCTCTCCTGCAAATCTGGGCC
Pol-probe	/56FAM/CACGTACTGGAGGAGGGTCACCTG
GAPDH-F	CCGCGATCTAATGTTCTCTTTC
GAPDH-R	TTCACTCCGACCTTCACCAT
GAPDH-probe	5Hex/CAGCCGCGTCCCTGAGACAC
RPA1-F	ACCCAGACGAACTGCTCAA
RPA1-R	TGGCGTCACTGATAGGTAAAT
RPA1-probe	/5Hex/TCACAGGCGTGGGCTTTCTGC
**Vector construction primers**	
AXM-124-pb-amp-S9-F	GGTCATCATCACCATCACCATTGAGTTTAA<br>ACCCCTAGAGCTCGCTGATCAGCCTCGAC
AXM-124-pb-amp-S9-R	CTTGAAAAAGTGGCACCGAGTCGGTGCTT<br>TTTTAAGCAGCTCCAGCCTACACAATCGCTC

AXM-125-SV40-neo-U6-F	ATAACTAGTCAATAATCAATGTCAACGCGT GGAACTCCATATATGGGCTATGAACTAATG
AXM-125-SV40-neo-U6-R	CTTGAGCGATTGTGTAGGCTGGAGCTGCTT AAAAAAGCACCGACTCGGTGCCACTTTTTC

Supplementary Table S2. Summary of base editing rate in porcine fetal fibroblast using BE3 system

Target gene	No. of clones	No. of mutants (%)	No. of Heterozygote (%)	No. of Homozygote (%)	No. of Indel (%)
TWIST2	43	36 (84)	25 (58)	11 (26)	1 (2)
TYR	66	8 (12)	7 (11)	1 (2)	2 (3)
PERV	326	303 (93)	NA	NA	NA

NA: not applicable.

Supplementary Table S3. Summary of base editing rate in offspring using BE3 system

Target gene	No. of offspring	Heterozygote (%)	Homozygote (%)	Indel (%)
TWIST2	21	0 (0)	21 (100)	0 (0)
TYR	8	4 (50)	3 (37.5)	1 (12.5)

Supplementary Table S4. Potential off-target sites of *TYR* and *TWIST2* sgRNAs in the pig genome

No.	Gene	Sequence	Chromosome	Position	Direction	Mismatches
TYR-On	*TYR*	ACCTCAGTTCCCCTTCACCG<u>GGG</u>	chr9	22517294	+	0
TYR-OT1	*CD79B*	ACCTCAGTgCCCCTTCcCCcAGG	chr12	15054293	+	3
TYR-OT2	*SHISA9*	ACCTCAGTTCCtCTTCACgtGGG	chr3	30075529	-	3
TYR-OT3	Intergenic region	ACCTtAaTTCCCCTTaACCGTGG	chr7	108719782	+	3
TYR-OT4	*TFB1M*	ACCTtAaTTtCCCTTCACCaTGG	chr1	11567676	+	4
TYR-OT5	*PKP4*	AtCTCtGTTCCCCTTCACCtTGG	chr15	65547995	+	3
TYR-OT6	*CDC123*	ACCTCAGcTCCCCTaCACCGTGG	chr10	59765083	-	3
TYR-OT7	Intergenic region	ACCcCAGTTCCCCTTCcCtGTGG	chr6	2093783	+	3
TYR-OT8	Intergenic region	ACCTCAGccCCCCTcCACtGGGG	chr1	103637384	+	4
TYR-OT9	Intergenic region	ttCTCAGTaCCCCTTCACCcAGG	chr1	104219258	+	4
TYR-OT10	*MYO1E*	ACCTCtaTTCCCCTcaACCGGGG	chr1	112765195	-	4
TWIST2-On	*TWIST2*	GGCGCTCGCGCACGTTGGCC<u>AGG</u>	chr15	138229016	-	0
TWIST2-OT1	*CDH13*	GGCtgTCGCGtACGTTtGCCTGG	chr6	5225147	-	4
TWIST2-OT2	Intergenic region	GGCGCTCGgcCACtTTGGCCTGG	chr11	6011018	-	3
TWIST2-OT3	Intergenic region	GGCGggCGtGCACGTTGGCCCGG	chr14	141044812	-	3

TWIST2-OT4	*TULP4*	GGCGCTgcgtCACGTTGGCCAGG	chr1	8655457	+	4
TWIST2-OT5	*GRK2*	GGCtgTCGCGtACGTTtGCCTGG	chr2	5225147	-	4
TWIST2-OT6	*PLIN2*	GGCGCTgGCGCAttTTtGCCGGG	chr1	203683855	+	4
TWIST2-OT7	*GARNL3*	GGCGCTCtCGCcgGTTGtCCTGG	chr1	267902925	+	4
TWIST2-OT8	*ITPRIPL2*	GGCtaTaGCGCACGgTGGCCAGG	chr3	26405411	-	4
TWIST2-OT9	*TRPM5*	GGCGCTCGaGggCGgTGGCCTGG	chr2	1646979	+	4
TWIST2-OT10	Intergenic region	GGgGCTgGCaCACGTTGGgCTGG	chr2	3758558	+	4

OT: Off-target

Supplementary Table S5. list of primers for amplifying and sequencing of off-target fragments

Primer Name	Primer sequence
TYR-OT1-F	CCCAACTTGTGTGGCTCTTGAGC
TYR-OT1-R	CCTTCCAGCTCCACCAAAGG
TYR-OT2-F	AGTCAAGATGCTGGCCAGGCT
TYR-OT2-R	TAAAAGCCCCGCCACCCTGC
TYR-OT3-F	AGCAACATCAGTCCGAGGGCT
TYR-OT3-R	TGGGAACTGTTGCGGGTAGA
TYR-OT4-F	TGAGTCTCTATTCACCTTCCCAAA
TYR-OT4-R	AGGACAGCTGTTCCACCTTAAACT
TYR-OT5-F	GGCACAGCTGGTCCCTAAC
TYR-OT5-R	TGAATAGGGCCCATCAGCTT
TYR-OT6-F	CAAGCTCACAGTCAATAAGAAACG
TYR-OT6-R	GGTTTCACACATTCTAAAGAAAGGG
TYR-OT7-F	GGTGCAGTGGTGGCGAGT
TYR-OT7-R	GACCCTTCCTGAGTCCACA
TYR-OT8-F	TCCTCACAGACTTTTGCATTAGGT
TYR-OT8-R	TCCGCTGGATGGTTCTGCTCTAAT
TYR-OT9-F	TGTCCGCCACCAAGACTGAGATAC
TYR-OT9-R	TGCCAGTCTCAAAAGGTTACATATGGT
TYR-OT10-F	TCATGTCTTGCTGGTGTTGTGAGA
TYR-OT10-R	CTGAAACAAGCACAGGTGGGTACT
TWIST2-OT1-F	AACTATTAAATGAGAAAATACCACCTGC
TWIST2-OT1-R	GACGCCGTCCTTTGCTTTGTG
TWIST2-OT2-F	GGGGAGGGAGCTTTTATTTCTGGT
TWIST2-OT2-R	ACTGGAGACTCTGGCCAACTTTCA
TWIST2-OT3-F	CTACCTGCACATGAAAAGCTGCCG
TWIST2-OT3-R	GACCCTGTCTCTCCCTCCCTGG
TWIST2-OT4-F	GCCCAAACAAGCAACAATGGAG
TWIST2-OT4-R	CAGTTTGGAAGGATCGACGGCAAC
TWIST2-OT5-F	ATGAGAAAATACCACCTGCCATGC
TWIST2-OT5-R	GGCCGTTCCTGCACCAAG
TWIST2-OT6-F	TTTAAAGACAGTTCTACTCCGAGGAC
TWIST2-OT6-R	CCCGAGGCCTCTTGAAATCCGAG
TWIST2-OT7-F	TCTTCACGTCGCTCTGCTCCTG
TWIST2-OT7-R	CCACCCATTTCAACGTGATCCCCA
TWIST2-OT8-F	AGACGGCTTCTGTGTGGATGTG

TWIST2-OT8-R	CAAATGGACGGCAGGGATAAGAC
TWIST2-OT9-F	GAAGGTGGGAGGAGGTTGGCG
TWIST2-OT9-R	CGTCAGGCTCTTGGAACTTCAGCA
TWIST2-OT10-F	CCTCCTCCTGGCATCCTGACTATACA
TWIST2-OT10-R	CCAGGAGGTGAACACGGCAGG

Supplementary Table S6. List of primers for illumina sequencing

Primer Name	Primer sequence
Pol-NGS3-F1-1	ACGAGACTGATTCCCTCCTCCAGTACGTGGATGACC
Pol-NGS3-F1-2	GCTGTACGGATTCCCTCCTCCAGTACGTGGATGACC
Pol-NGS3-F1-3	ATCACCAGGTGTCCCTCCTCCAGTACGTGGATGACC
Pol-NGS3-F1-4	TGGTCAACGATACCCTCCTCCAGTACGTGGATGACC
Pol-NGS3-F1-5	ATCGCACAGTAACCCTCCTCCAGTACGTGGATGACC
Pol-NGS3-F1-6	GTCGTGTAGCCTCCCTCCTCCAGTACGTGGATGACC
Pol-NGS3-F1-7	AGCGGAGGTTAGCCCTCCTCCAGTACGTGGATGACC
Pol-NGS3-F1-8	ATCCTTTGGTTCCCCTCCTCCAGTACGTGGATGACC
Pol-NGS3-F1-9	TACAGCGCATACCCCTCCTCCAGTACGTGGATGACC
Pol-NGS3-F1-10	ACCGGTATGTACCCCTCCTCCAGTACGTGGATGACC
Pol-NGS3-F1-11	AATTGTGTCGGACCCTCCTCCAGTACGTGGATGACC
Pol-NGS3-F1-12	TGCATACACTGGCCCTCCTCCAGTACGTGGATGACC
Pol-NGS3-F1-13	AGTCGAACGAGGCCCTCCTCCAGTACGTGGATGACC
Pol-NGS3-F1-14	ACCAGTGACTCACCCTCCTCCAGTACGTGGATGACC
Pol-NGS3-F1-15	GAATACCAAGTCCCCTCCTCCAGTACGTGGATGACC
Pol-NGS3-F2-1	ACGAGACTGATTCCCTCCTCCAGTTCGAGGATGACC
Pol-NGS3-F2-2	GCTGTACGGATTCCCTCCTCCAGTTCGAGGATGACC
Pol-NGS3-F2-3	ATCACCAGGTGTCCCTCCTCCAGTTCGAGGATGACC
Pol-NGS3-F2-4	TGGTCAACGATACCCTCCTCCAGTTCGAGGATGACC
Pol-NGS3-F2-5	ATCGCACAGTAACCCTCCTCCAGTTCGAGGATGACC
Pol-NGS3-F2-6	GTCGTGTAGCCTCCCTCCTCCAGTTCGAGGATGACC
Pol-NGS3-F2-7	AGCGGAGGTTAGCCCTCCTCCAGTTCGAGGATGACC
Pol-NGS3-F2-8	ATCCTTTGGTTCCCCTCCTCCAGTTCGAGGATGACC
Pol-NGS3-F2-9	TACAGCGCATACCCCTCCTCCAGTTCGAGGATGACC
Pol-NGS3-F2-10	ACCGGTATGTACCCCTCCTCCAGTTCGAGGATGACC
Pol-NGS3-F2-11	AATTGTGTCGGACCCTCCTCCAGTTCGAGGATGACC
Pol-NGS3-F2-12	TGCATACACTGGCCCTCCTCCAGTTCGAGGATGACC
Pol-NGS3-F2-13	AGTCGAACGAGGCCCTCCTCCAGTTCGAGGATGACC
Pol-NGS3-F2-14	ACCAGTGACTCACCCTCCTCCAGTTCGAGGATGACC
Pol-NGS3-F2-15	GAATACCAAGTCCCCTCCTCCAGTTCGAGGATGACC
Pol-NGS5-R1-1	ACGAGACTGATTTCCAGCTGTCCCCAAAAACTCTCT
Pol-NGS5-R1-2	GCTGTACGGATTTCCAGCTGTCCCCAAAAACTCTCT
Pol-NGS5-R1-3	ATCACCAGGTGTTCCAGCTGTCCCCAAAAACTCTCT
Pol-NGS5-R1-4	TGGTCAACGATATCCAGCTGTCCCCAAAAACTCTCT
Pol-NGS5-R1-5	ATCGCACAGTAATCCAGCTGTCCCCAAAAACTCTCT
Pol-NGS5-R1-6	GTCGTGTAGCCTTCCAGCTGTCCCCAAAAACTCTCT
Pol-NGS5-R1-7	AGCGGAGGTTAGTCCAGCTGTCCCCAAAAACTCTCT

Pol-NGS5-R1-8	ATCCTTTGGTTCTCCAGCTGTCCCCAAAAACTCTCT
Pol-NGS5-R1-9	TACAGCGCATACTCCAGCTGTCCCCAAAAACTCTCT
Pol-NGS5-R1-10	ACCGGTATGTACTCCAGCTGTCCCCAAAAACTCTCT
Pol-NGS5-R1-11	AATTGTGTCGGATCCAGCTGTCCCCAAAAACTCTCT
Pol-NGS5-R1-12	TGCATACACTGGTCCAGCTGTCCCCAAAAACTCTCT
Pol-NGS5-R1-13	AGTCGAACGAGGTCCAGCTGTCCCCAAAAACTCTCT
Pol-NGS5-R1-14	ACCAGTGACTCATCCAGCTGTCCCCAAAAACTCTCT
Pol-NGS5-R1-15	GAATACCAAGTCTCCAGCTGTCCCCAAAAACTCTCT
